# Clinical Use of Anti-Xa Monitoring in Malignancy-Associated Thrombosis

**DOI:** 10.1155/2015/126975

**Published:** 2015-10-12

**Authors:** Sarah Yentz, Oluwatoyosi A. Onwuemene, Brady L. Stein, Elizabeth H. Cull, Brandon McMahon

**Affiliations:** ^1^Northwestern University Feinberg School of Medicine, Chicago, IL 60611, USA; ^2^Department of Medicine, Duke University, Durham, NC 27710, USA

## Abstract

*Introduction*. Low molecular weight heparin (LMWH) is preferred for malignancy-associated venous thromboembolism (VTE). Many providers monitor LMWH with anti-Xa levels, despite little validation on correspondence with patient outcome.* Methods*. This is a retrospective, single institution study of anti-Xa measurement in malignancy-associated thrombosis. Cases were identified using the Electronic Data Warehouse, and inclusion was confirmed by two independent reviewers. Malignancy type, thrombotic history, measurement rationale and accuracy, clinical context, and management changes were evaluated.* Results*. 167 cases met inclusion criteria. There was no clear rationale for anti-Xa testing in 56%. Impaired renal function (10%), documented or suspected recurrent thrombosis despite anticoagulation (9%), and bleeding (6%) were the most common reasons for testing. Incorrect measurement occurred in 44%. Renal impairment was not a significant impetus for testing, as 70% had a GFR > 60. BMI > 30 was present in 40%, and 28% had a BMI < 25. Clinical impact was low, as only 11% of patients had management changes.* Conclusions*. Provider education in accuracy and rationale for anti-Xa testing is needed. Our study illustrates uncertainty of interpretation and clinical impact of routine anti-Xa testing, as management was affected in few patients. It is not yet clear in which clinical context providers should send anti-Xa levels.

## 1. Introduction

Since the CLOT study [1], low molecular weight heparin (LMWH) has been the preferred treatment for malignancy-associated venous thromboembolism (VTE) given its improved efficacy compared to warfarin. There has been a steady increase in the use of LMWH for this indication. A recent study of malignancy associated VTE showed that rates of LMWH use increased from 18% in 2000 to 31% in 2007 [2]. The more consistent anticoagulant effect seen with LMWH is another advantage over warfarin. The biologic variability associated with LMWH treatment is highly reproducible [3] and has been shown to have greater than 90% bioavailability [4]. Typically, LMWH therefore does not routinely require monitoring, but anti-Xa levels can be used for this purpose.

Measurement and interpretation of anti-Xa levels in LMWH use can be complicated. In contrast to the INR, an anti-Xa level must be collected as a peak, approximately 4 hours after at least the third dose of LMWH. The time dependency of the test makes it more difficult to perform, and inaccurate timing of collection may result in inappropriate evaluation of coagulation status. Even when checked correctly, there is little clinical validation on how anti-Xa levels should be interpreted and how they correspond with patient outcome, if at all. Despite published “therapeutic” ranges, they have not been shown in clinical studies to reflect improved efficacy compared to subtherapeutic levels, or improved safety compared to higher levels [5, 6]. There is therefore no clear consensus on the therapeutic range for anti-Xa activity in patients receiving prophylactic or treatment doses of LMWH for either VTE or acute coronary syndrome indications [7].

Although there is little clinical validation for use of anti-Xa levels, it is a commonly used laboratory test among practitioners. The concern for a need to monitor often relates to uncertainty regarding how LMWH behaves in specific populations, particularly those with renal insufficiency, extremes of body weight, and pregnancy. Additionally, despite improved outcomes over warfarin, patients with malignancy-associated VTE treated with LMWH still have a 9% risk of recurrent thrombosis at 6 months [1]. There has been little evidence on whether anti-Xa levels impact the potential for recurrence. Even if there is a relationship, optimal management of these recurrent events remains unclear with a paucity of clinical studies in this area.

To better understand how anti-Xa levels are incorporated into clinical practice, we conducted a retrospective review of patients with malignancy associated VTE who had an anti-Xa level checked during their hospitalization at our institution. The goal was to evaluate whether the anti-Xa level was checked correctly, the impetus for checking the level, what portion of levels drawn were therapeutic, and whether results impacted management.

## 2. Methods

**Figure 1:**
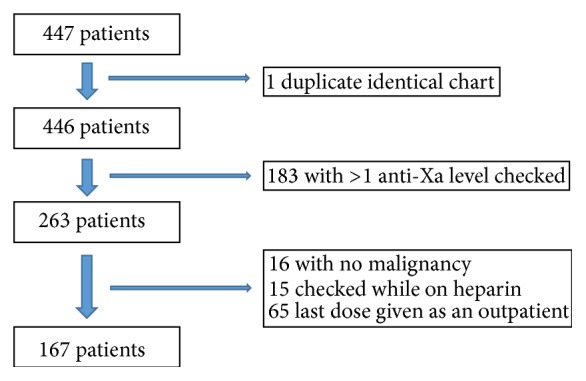
Patient characteristics of the study population.

This is a single-institution, retrospective study. Cases of malignancy-associated thrombosis occurring between 1/1/2006 and 12/31/2011 were identified using current procedural terminology (CPT) codes and the Northwestern University Electronic Data Warehouse. This cohort was then screened for those who had a LMWH anti-Xa level checked at any point in that time frame. All resultant cases had charts reviewed by 2 independent investigators to confirm malignancy, treatment with LMWH, and history of VTE. Of those cases, only those with one anti-Xa level checked were included in the current analysis. Patients were excluded from analysis if the anti-Xa level was checked while on unfractionated heparin and if the last dose of anticoagulation was given as an outpatient (Figure 1).

Charts were evaluated for demographic data, thrombotic history, reasoning and accuracy of anti-Xa level, events leading up to the anti-Xa level being drawn, and changes in management after an anti-Xa level resulted. An anti-Xa level was considered to be checked correctly if the level was drawn 4–6 hours after LMWH administration and only after the patient had received ≥3 consecutive doses of LMWH. A therapeutic anti-Xa level for dalteparin was 0.5–1.5 IU/mL. A therapeutic anti-Xa level for enoxaparin was 0.5–1.0 IU/mL. Levels falling above or below these ranges were considered supratherapeutic and subtherapeutic, respectively. If the dose of LMWH was changed, or there was a switch in the type of anticoagulation given within the 24–48 hours after the anti-Xa level was drawn, this was considered a change in management.

## 3. Results

After exclusions, the data from 167 patients was analyzed. Patient characteristics are represented in Table 1. The mean age of the cohort was 65.2 years, with slightly more men than women included. Dalteparin was used in the majority of cases. Despite being on Coumadin (*n* = 1) and fondaparinux (*n* = 7), a LMWH anti-Xa level was drawn in these patients (Table 1). Two patients with malignancy-associated VTE had anti-Xa levels checked while not actively receiving anticoagulation. Hematologic malignancies and lung and gastrointestinal cancers together accounted for about half of the evaluated cases (Table 1). Objective documentation of renal function was available in 164 patients. Body mass index (BMI) was documented or able to be pulled from 141 patient charts.

### 3.1. Rationale for Testing

**Figure 2:**
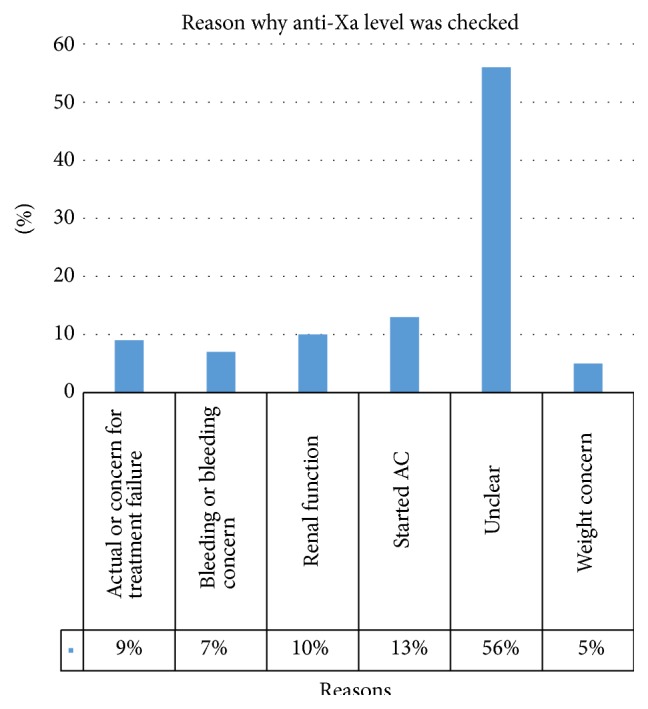
Reason providers checked an anti-Xa level. Those patients where the reason was not documented and the patient failed to otherwise fit into other categories were counted as “unclear.” AC: anticoagulation.

We did a comprehensive chart review to evaluate why the physician decided to order an anti-Xa level in each patient. Despite careful review of the medical record, a reason could not be identified in 56% (94/167) of patients. The most common identifiable reason was a new start of anticoagulation (13%), followed by concerns about renal function (10%), actual or concern for treatment failure (9%), bleeding or bleeding concern (7%), and patient weight (5%) (Figure 2).

### 3.2. Accuracy of Lab Test

Out of 167 patients, only 56% (94/167) had their anti-Xa level checked correctly. In those patients with an anti-Xa level checked correctly, 75% (64/85) were considered therapeutic. All (100%) of the remaining 21 patients with an accurately checked level who fell outside of the reference range were considered subtherapeutic. None of the correctly checked labs were at a supratherapeutic level.

### 3.3. Renal Function

**Figure 3:**
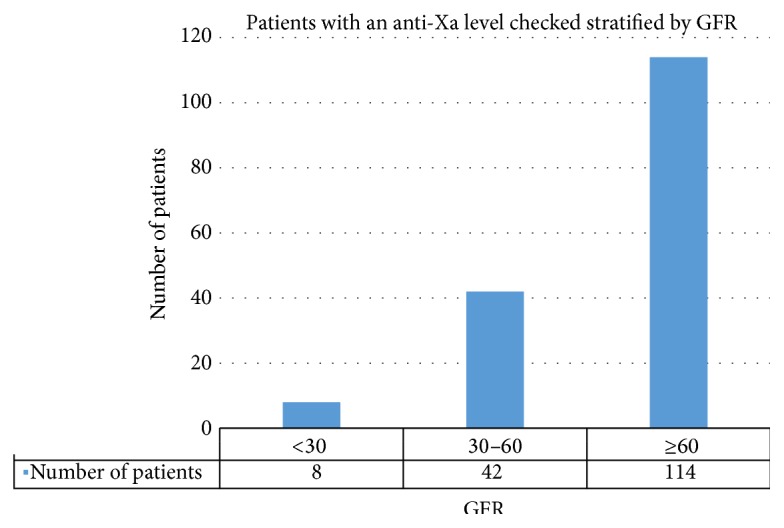
Analysis of those patients who had the anti-Xa level checked correctly, stratified by GFR. Presence or absence of renal impairment did not influence whether an anti-Xa level was in the therapeutic range. 27% of patients with a “normal” GFR were still found to have a subtherapeutic anti-Xa result.

114 of the 164 patients (114/164, 70%) evaluated had a GFR > 60. Impaired creatinine clearance did not appear to be a strong driver for practitioners to check an anti-Xa level as there were very few patients with a GFR < 30 (8/164, 5%). Those patients with correctly checked levels were further divided by GFR (Figure 3). There was not a correlation between GFR and whether anti-Xa levels were therapeutic, noting that there were fewer patients with a GFR < 60 in the analysis. However, of patients with a normal GFR, 27% (17/64) were found to be subtherapeutic.

### 3.4. BMI

**Figure 4:**
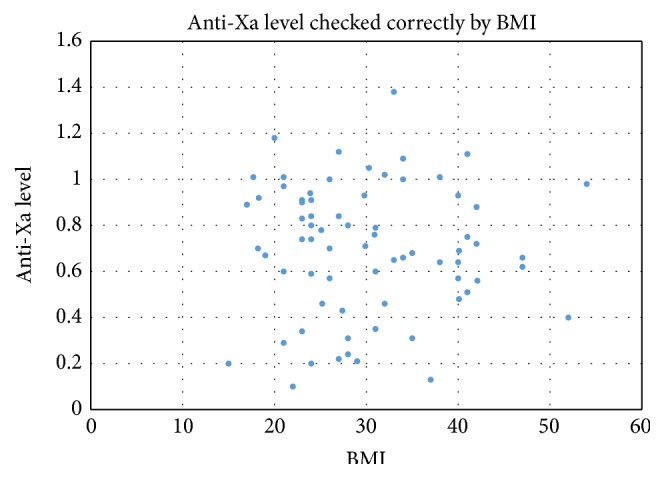
In patients who were on therapeutically dosed LMWH and had a level checked correctly (*n* = 71), there was no correlation between BMI and resultant anti-Xa.

When anti-Xa levels were stratified by BMI, 40% of the patients who had an anti-Xa level checked had a BMI > 30 (57/141) compared to 28% (39/141) of patients with a normal BMI (18.5–24.9). Obese patients were just as likely as normal weight patients to have a therapeutic anti-Xa level. In patients with normal BMIs, 79% (15/19) of patients whose anti-Xas were checked correctly were therapeutic compared with 81% (25/31) of patients with obese BMIs. There was no correlation between the BMI and the anti-Xa level in those patients who had an anti-Xa level checked correctly (Figure 4).

### 3.5. Management

Charts were further evaluated for management decisions after an anti-Xa level was drawn. Assessing how the result (therapeutic or not) could potentially influence decision making was difficult, since rationale for testing was not evident in more than half the cases. In total, only 11% (19/167) of patients had any change in management. Dose adjustment in current regimen was the most common change (17/19), with only 2 instances of switching to an alternative agent. Interestingly, of patients with a change in management, 53% (10/19) had an anti-Xa level that was drawn incorrectly.

## 4. Discussion

Despite widespread use in routine clinical practice, the role for checking LMWH anti-Xa levels in patients with malignancy-associated thrombosis is not clear. Our data indicate that providers may not be aware of how the lab should be drawn, with regard to peak levels. Providers also appear unaware of the indications for checking an anti-Xa level. The most common identifiable reason was a new start of anticoagulation. From this reason, one can infer the physician was uncomfortable with the standard weight based dose and wanted a lab test to “prove” correct dosing. In addition, the majority of anti-Xa levels were checked in overweight and obese patients, despite no definitive evidence that these patients require extra monitoring [8, 9].

As the medical field becomes increasingly aware of cost, it is clear that further education of providers is required regarding potential indications for checking an anti-Xa, accurate timing of laboratory testing, and interpretation of results. Over half of our cohort had no clear indication for testing, and almost half had the level drawn incorrectly, rendering the result useless. At our hospital, an anti-Xa level costs $94. In our sample population, nearly $7000 was spent on anti-Xa levels that were not checked correctly.

There has been a misconception in clinical practice that obese patients treated with LMWH require anti-Xa monitoring. This misconception arose from concerns that using actual instead of ideal weight based dosing of LMWH would lead to supratherapeutic levels of anticoagulation in obese patients as LMWH is distributed to just the intravascular space and not to adipose tissue. A study by Wilson et al. found similar anti-Xa levels in patients within 20% of ideal body weight and those >40% of ideal body weight, concluding that dosing should be based on actual, not ideal body weight and that dose capping was unnecessary [8]. Several additional studies have addressed the question of whether BMI influences anti-Xa levels. They too have found no difference in anti-Xa levels in patients with normal versus elevated BMIs [9, 10]. Our study is in line with these prior evaluations. Our results demonstrate no correlation between an elevated BMI and either sub- or supratherapeutic anti-Xa levels.

Although there is little role for monitoring anti-Xa levels in obese patients, there may be a role for anti-Xa monitoring in severe renal insufficiency. Several studies have shown that impaired creatinine clearance leads to higher anti-Xa levels [9, 11]. The meta-analysis by Lim et al. showed that use of LMWH in patients with a creatinine clearance of 30 mL/min or less was associated with an increased risk for major bleeding. In our study, impaired creatinine clearance did not seem to be a major impetus for checking a level as 70% of the anti-Xa levels were checked in patients with a GFR > 60. Numbers of patients with low GFR (<30) were too small in the present study to evaluate whether there was indeed a correlation with a higher anti-Xa level and hence potential propensity for bleeding complications.

Further work should be done to better clarify the role for anti-Xa levels, starting with an improved definition of the therapeutic range. Currently, the therapeutic ranges are based upon expert opinion [12] rather than definitive evidence that correlates anti-Xa levels to patient outcomes. A study using the American Pathologist therapeutic range found that 50% of patients with normal weight and renal function did not achieve anti-Xa levels considered “therapeutic” [9]. The study did not comment on outcomes of these patients; however it is unlikely that all 50% went on to develop further thrombosis or bleeding.

Our study shows that despite commonly ordering anti-Xa levels, providers are infrequently using them to make management decisions. Of the 167 patients who had a level drawn, only 19 had a change in dose or type of LMWH after the level resulted and 20 had a correctly drawn anti-Xa that was subtherapeutic. This brings into question the rationale for checking the level initially and how to best interpret those results to apply to specific clinical scenarios. For example, a potential role for anti-Xa monitoring could be in those patients who experience a recurrent thrombotic event despite seemingly adequate anticoagulation. This is a relatively common occurrence in those with malignancy-associated thrombosis. Dose escalation of LMWH may be indicated in those patients with levels below or at the lower end of the reference range. However, more clinical data would be required to support this, and more evidence is needed to justify what is considered the therapeutic range.

Our study had several limitations. First, it was a small, single institution study. Although all of our patients had a malignancy, not all patients were actively receiving chemotherapy, a known and added risk factor for VTE in cancer. The retrospective nature limited investigation of the reasoning behind checking an anti-Xa level as the thought process was not always clearly delineated in documentation.

In summary, our results strongly suggest a role for provider education if testing is felt to be clinically indicated. At present, though testing is widely available, it is not yet clear in which clinical contexts (if any) providers should send LMWH anti-Xa levels. Our study, like others before it [8, 9] showed no correlation between anti-Xa levels and BMI; hence there is limited value in monitoring anti-Xa levels in obese patients. Prospective studies are needed to better clarify whether anti-Xa levels correlate with clinical outcomes in those with malignancy-associated thrombosis and how the results should be incorporated clinical practice.

## Figures and Tables

**Table 1:** Patient characteristics.

Cohort characteristics	Number	%
Age distribution		
20–30	5	3%
31–40	6	4%
41–50	15	9%
51–60	34	20%
61–70	40	24%
71–80	38	23%
81–90	24	14%
>90	5	3%
Mean age	65.2	
Gender		
Male	86	51.50%
Female	81	48.50%
Distribution by malignancy		
Breast	15	9%
GI	19	11%
Gyn	10	6%
Colorectal	14	8%
Lung	23	14%
Hematologic	41	25%
Prostate	13	8%
Renal cell	5	3%
Skin	3	2%
Urothelial	8	5%
Head/neck	6	4%
Other	10	6%
Type of anticoagulation		
Coumadin	1	1%
Dalteparin	152	91%
Enoxaparin	5	3%
Fondaparinux	7	4%
None	2	1%
